# How HIV/AIDS scale-up has impacted on non- HIV priority services in Zambia

**DOI:** 10.1186/1471-2458-10-540

**Published:** 2010-09-08

**Authors:** Ruairí Brugha, Joseph Simbaya, Aisling Walsh, Patrick Dicker, Phillimon Ndubani

**Affiliations:** 1 Departmemt of Epidemiology and Public Health Medicine, Division of Population Health Sciences Division, Royal College of Surgeons in Ireland, Dublin, Ireland; 2 Institute of Economic and Social Research, University of Zambia, Lusaka, Zambia; 3 Department of Global Health Development, Faculty of Public Health and Policy, London School of Hygiene and Tropical Medicine, London, UK

## Abstract

**Background:**

Much of the debate as to whether or not the scaling up of HIV service delivery in Africa benefits non-HIV priority services has focused on the use of nationally aggregated data. This paper analyses and presents routine health facility record data to show trend correlations across priority services.

**Methods:**

Review of district office and health facility client records for 39 health facilities in three districts of Zambia, covering four consecutive years (2004-07). Intra-facility analyses were conducted, service and coverage trends assessed and rank correlations between services measured to compare service trends within facilities.

**Results:**

VCT, ART and PMTCT client numbers and coverage levels increased rapidly. There were some strong positive correlations in trends within facilities between reproductive health services (family planning and antenatal care) and ART and PMTCT, with Spearman rank correlations ranging from 0.33 to 0.83. Childhood immunisation coverage also increased. Stock-outs of important drugs for non-HIV priority services were significantly more frequent than were stock-outs of antiretroviral drugs.

**Conclusions:**

The analysis shows scale-up in reproductive health service numbers in the same facilities where HIV services were scaling up. While district childhood immunisations increased overall, this did not necessarily occur in facility catchment areas where HIV service scale-up occurred. The paper demonstrates an approach for comparing correlation trends across different services, using routine health facility information. Larger samples and explanatory studies are needed to understand the client, facility and health systems factors that contribute to positive and negative synergies between priority services.

## Background

Funding for HIV and AIDS control in Africa is heavily reliant on a small number of donors, with external funding in 2006 estimated to have accounted for between 67% and 81% of all AIDS funding in four African countries [1]. Zambia was in the mid range at 74% donor-reliant. Almost half (49%) of all external funding for HIV/AIDS came from two global health initiatives (GHIs), which specifically targeted HIV and AIDS [2]: The Global Fund to Fight AIDS, Tuberculosis and Malaria and the United States President's Emergency Plan for AIDS Relief (PEPFAR). Globally, disbursements for HIV control grew from just over 3% of development assistance for health in 1990 to 23% [3] or 47% in 2007 [4], depending on the data source. While funding to HIV and AIDS has been generally welcomed - and with unvarnished enthusiasm by some - others have expressed concern about the possible effects of HIV/AIDS funding levels that reportedly exceed the total amount allocated to the health sector in some African countries [2,5,6].

Much of the debate has been around displacement and crowding-out effects that spending on HIV and AIDS might have on other health and development priorities [4,6], with claims of negative effects through a shift of funding away from health systems strengthening [4,5]; and from other non-HIV/AIDS disease control priorities [7,8]. Counter-claims that spending on HIV/AIDS strengthens health systems have been made, often by those who work in the field of HIV/AIDS control [9,10]. Justification given for HIV spending is that HIV disease over-burdens health facilities, crowds out care for other conditions and the introduction of antiretroviral treatment (ART) reduces the AIDS burden at health facilities [10,11]. Mechanisms have been suggested whereby HIV funding would strengthen laboratory systems [12] and pharmacy management [13], and lead to improvements in the delivery of other disease control programmes. However, there is limited empirical evidence on the impact of HIV scale-up on the routine delivery of other priority services and "opinion rather than evidence has dominated the debate" [14]. While drafting this paper, we found one paper that reported positive effects, whereby syphilis screening of pregnant women increased significantly, in association with Prevention of Mother to Child HIV Transmission (PMTCT) scale-up [15].

Large amounts of Global Fund and PEPFAR money were flowing into Zambia from 2004 and significant scale-up of ART and PMTCT was taking place [1,16,17]. We conducted a study to explore the health systems effects of this scale-up, specifically in the area of human resources for health (reported elsewhere) and on other non-HIV priority service delivery and coverage. This paper presents and discusses correlations in intra-facility trends in client numbers and population coverage for a range of HIV and non-HIV priorities. It does this by analysing retrospective health facility record data, 2004-07, across a range of public and NGO health facilities in Zambia. The paper aims to demonstrate the utility of analysing routine health facility data, and proposes further research that could explain service trends and alert programme managers to areas that might require responses.

## Methods

Three districts were purposively selected for the study to represent the capital city (Lusaka), an urban district (Kabwe) and a rural district (Mumbwa). District health facilities were mapped in 2007 and again in 2008 to identify all fixed government and NGO facilities providing HIV or AIDS services (those providing outreach services only and Ministry of Defence and private for-profit facilities were excluded). Based on discussions with District Health Management Teams (DHMTs), 39 facilities were selected for the survey (n = 33 government and n = 6 NGO/mission). These included all 29 facilities that reported delivering ART (24 government and 5 NGO/mission). The sample also included a purposive sample of 10 facilities that were reported by the DHMTs as important providers of HIV services, though not ART (1 facility in Lusaka, 2 in Kabwe and 7 in Mumbwa). All district, mission and central hospitals, and the University Teaching Hospital (UTH) in Lusaka, were included. Ethics approval for the study was granted by the University of Zambia Research Ethics Committee.

Following a pilot survey, a trained and supervised team of field workers extracted and recorded facility record data in June-July 2008, including patient/client and service episode records for complete years 2004-2007 inclusive. The proformas used to record the facility record data, which provide the basis for this paper, were adapted based on lessons learned from a similar February 2007 facility survey. Non-HIV patient record data that were collected from facilities were supplemented by electronic summaries of facility record-return data kept at district health offices. Where there were two sources of data, the most complete data set was used in the analysis. For example, district offices supplied complete data on family planning and antenatal clinic clients for 29 facilities from 2004 to 2007, compared to 21 facilities whose records' departments had complete data on family planning and 19 on antenatal client registrations. The most complete data held at district offices were for reproductive health (family planning and antenatal care) and childhood immunisations, which made them the most useful data for estimating non-HIV performance. HIV service data were not available from district offices in Zambia and were collected directly from the facilities that were delivering ART, Voluntary Counselling and Testing (VCT) or PMTCT services.

Quantitative data were entered, cleaned and analysed using SPSS (Version 16.0), with further analysis using SAS (Version 9.1). Where facility data were missing for one time period within a trend analysis, this facility was omitted from the analysis, which reduced the numbers of units in some analyses.

## Results

Table 1 summarises the range of HIV related services identified in the 2008 mapping exercise: all 39 health facilities offered VCT services, almost all (37) provided PMTCT, 29 provided ART; and most offered a wide range of HIV support services. Of facilities that provided HIV services, significantly fewer in rural Mumbwa - 5/11 (45%) - provided ART compared to the two urban districts (p. < 0.01). Almost all (95%) of the facilities provided condoms. All facilities in Lusaka provided fee exemptions for people living with HIV/AIDS, compared with 90% in Kabwe and 91% in Mumbwa. Similarly, the provision of food and nutrition support was more widespread in Lusaka (100%) than in the other two districts and was significantly higher in urban than in rural facilities (31% in Mumbwa, p < 0.01). Home based care was provided by three quarters of facilities, with no significant difference by district or urban/rural setting.

**Table 1:** HIV and selected non-HIV services provided by district fixed facilities

	All Districts (n = 39)	Lusaka (urban) (n = 18)	Kabwe (urban) (n = 10)	Mumbwa (rural) (n = 11)	Urban (n = 28)	Rural (n = 11)
***Core HIV services***						

ART	29	16	8	5	24	5

VCT	39	18	10	11	28	11

PMTCT	37	16	10	11	26	11

***HIV support services***						

Food/nutrition support for HIV+ people	27	18	5	4	23	4

Income Generating activities for HIV+ people	13	8	1	4	9	4

Fee exemptions for people infected with HIV+	37	18	9	10	27	10

Information, Eductional and Communication materials	38	18	9	11	27	11

Home Based Care to HIV+ people	31	14	7	10	21	10

Spiritual Support to HIV+ people	23	10	4	9	14	9

n = numbers of facilities

**Figure 1:**
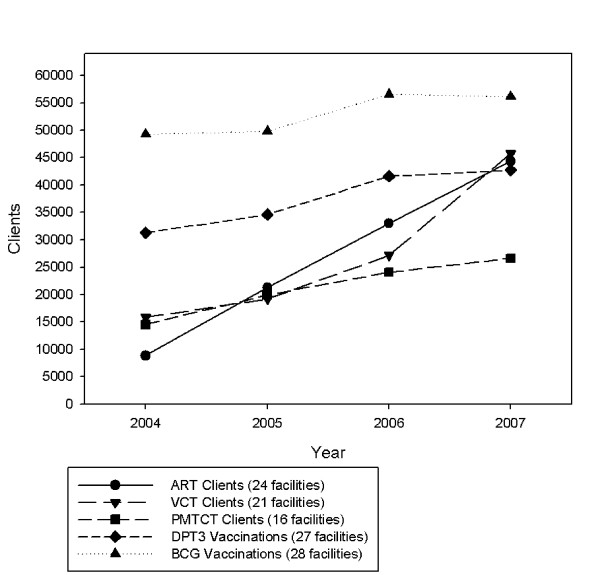
**Scale-up in HIV services and infant vaccinations, 2004-2007**.

Figure 1 shows the trends and scale-up in the numbers of clients who registered for ART, PMTCT, VCT, and the numbers of infants who received two important vaccinations across 2004-07, based on facilities that reported complete data across the four time periods. Numbers of clients on ART increased from 8,843 in 2004 to 44,311 in 2007; VCT clients rose from 15,838 to 45,777; and PMTCT clients from 14,541 to 26,066 in 2007. ART clients registered at the 24 facilities included in the analysis for this study constituted a sizable proportion of the national figures reported by Zambia in its 2008 UN General Assembly on AIDS (UNGASS) report [18]: 21,267 (54% of) clients reported nationally for 2005, 33,016 (41%) for 2006 and 44,311 (30%) of all clients reported to be on ART in Zambia in 2007. The high proportion of ART clients included in our study that were reported nationally was because we surveyed the main ART facilities in Lusaka, where scale-up started earlier. As scale-up rolled out to towns in 2006 and rural areas in 2007, there was a reduction in the proportion of clients receiving their ART in facilities in the capital city, both in our study and nationally.

Children's vaccinations (numbers of antigens given and coverage levels) provide useful indicators of the performance of non-HIV/AIDS services. Bacille Calmette Guerin (BCG), which reduces the risk of serious TB complications in children and is given as a long term measure to reduce TB in the population, is a good indicator of the number and proportion of infants starting immunisations. DPT 3 (the third antigen or dose of the 3-in-1 vaccine for Diphtheria, Pertussis and Tetanus), which requires that the child be vaccinated on three separate occasions, is a good indicator of the performance of the Expanded Programme of Immunisation. The third dose is given ideally before six months of age. The most complete immunisation data sets that were obtained from the DHMT were for BCG and DPT3, where complete data from 2004 to 2007 were obtained for 28 facilities (BCG) and 27 facilities (DPT3) delivering mother and child health services. Figure 1 shows a steady rise in the numbers of DPT 3 (31,290 to 44,311) and BCG doses (49,261 to 56,107) delivered annually, 2004 to 2007.

Table 2 shows that average ART coverage levels across the three districts increased steadily from 20% in 2004 to 75% in 2007 in the 16 facilities that had catchment populations and that were reporting complete data from 2004 to 2007. Facilities with missing data or reporting no ART provision across the four years were excluded from the analysis. The graph and table show a correction for 2007, including ART coverage for 18 surveyed facilities, including two additonal ones with catchment populations that reported ART delivery in 2007 alone. Coverage was estimated as follows: HIV prevalence estimates reported annually for Lusaka province, Central province urban (Kabwe) and rural (Mumbwa) were applied to facility catchment population estimates, adjusted for annual population growth, to estimate HIV positive catchment populations. Proportions of HIV positives (adults and children) with advanced disease and requiring ART were derived from those reported by Zambia for 2006 and 2007, projected back to 2005 and 2004 [18].

**Table 2:** ART coverage (those in need of ART) by district location, 2004-07

Location	Year	Numbers of facilities	Patients in need of ART	Patients receiving ART
Lusaka	2004	8	22315	5018 (22.5%)
	2005	8	23962	12898 (53.8%)
	2006	8	25711	19178 (74.6%)
	2007	8	27461	20577 (74.9%)
	2007*	10	36367	23549 (64.8%)
Kabwe	2004	4	2041	0 (0.0%)
	2005	4	2153	215 (10.0%)
	2006	4	2260	2734 (121.0%)
	2007	4	2360	2005 (85.0%)
	2007*	4	2360	2005 (85.0%)
Mumbwa	2004	4	504	0 (0.0%)
	2005	4	542	0 (10.0%)
	2006	4	580	35 (6.0%)
	2007	4	612	229 (37.4%)
	2007*	4	612	229 (37.4%)
Total	2004	16	24861	5018 (20.2%)
	2005	16	26657	13113 (49.2%)
	2006	16	28551	21947 (76.9%)
	2007	16	30433	22811 (75.0%)
	2007*	18	39340	25783 (65.5%)

* Note: 16 facilities reported continuous data across 2004-07 and a further two reported only in 2007.

An ART pilot had been launched in Lusaka in 2002 by the Ministry of Health in two large hospitals, but client load and coverage only began to increase significantly in 2004 with the support of the Global Fund and PEPFAR [16]. Scale-up in ART numbers and coverage levels started in earnest in Kabwe in 2005 and in rural Mumbwa in 2006 (Table 2). ART coverage percentages are likely to be an over-estimate of population coverage - Zambia reported national ART coverage at 33% for 2006 and 50% for 2007. The most plausible reason for higher coverage levels in our study is that some of those in need of ART were likely to have come from other catchment areas to visit the ART-providing facilities included in this analysis. ART scale-up in Lusaka from 2002 is likely to have attracted some of those who knew or suspected they needed ART. Total numbers and coverage percentages for Lusaka and the three districts combined are very similar, reflecting the contribution of the much larger population of ART clients in Lusaka.

**Figure 2:**
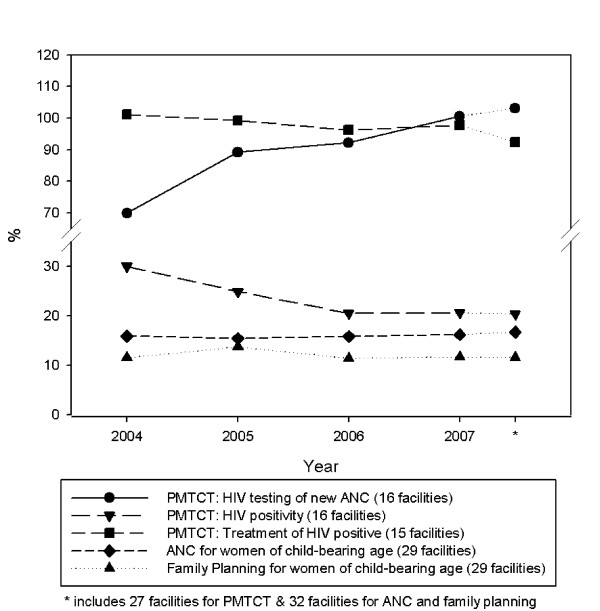
**PMTCT and reproductive health coverage, 2004-2007**.

In the 16 facilities providing PMTCT for which there were complete data across 2004-07 (Figure 1), the numbers of pregnant women attending for antenatal care who received a HIV test rose from 14,541 (2004) to 26,066 (2007). A similar rolling-out pattern to that of ART was seen. By 2004, 74% of pregnant women in PMTCT-providing facilities in Lusaka were already being tested, whereas in the six PMTCT facilities in rural Mumbwa, testing only began to rise in 2006 where it reached 36%, up from 3% in 2005, and quickly reached 89% by 2007. Again, health- (ART-) seeking behaviour, where clients came from other catchment areas, may have contributed to high coverage levels at these facilities. Figure 2 illustrates PMTCT programme trends in facilities with complete data for HIV testing (16 facilities), test results (16 facilities) and treatment (15 facilities). The percentage of antenatal clinic registrants who received a HIV test in these facilities rose from 70% in 2004 to 101% (100.6%) in 2007; the proportion of pregnant women who tested positive fell from 24% to 20%, and close to 100% (97.8%) of HIV test positive pregnant women were reported to have received antiretroviral treatment to prevent PMTCT.

The aim of this paper is to illustrate how non-HIV service delivery and population coverage were performing in districts where there was rapid scaling up in the delivery and coverage of HIV services. Figure 2 also includes Reproductive Health Service coverage 2004-07, calculated as the percentage of women of child-bearing age (WCA) (15-49 years) registered for antenatal care (ANC) and family planning (FP). Data on total numbers of antenatal visits were available, but health informations systems at the facilities and district office did not provide data on the numbers of women who completed four antenatal care visits; nor on numbers of family planning visits and adherence. Catchment population sizes based on census estimates and adjusted annually for population growth were used to calculate the target population. Antenatal client numbers increased steadily and coverage levels for both services were maintained across all 29 facilities that reported data consistently across the four years.

Target population sizes for immunisations were the number of children under one year of age in each health facility's catchment population, adjusted for population growth. This was a figure routinely reported by the DHMT. Overall, DPT3 coverage rates increased steadily, year-on-year, from 73% (2004) to 91% (2007) and without large fluctuations, supporting the hypothesis of an effective immunisation programme. Between 2004 and 2007, BCG coverage rates rose from 112% to 117%. The most likely explanation for BCG coverage rates greater than 100% is that BCG is given soon after birth of the child, usually at the first post-natal checkup visit. An unknown number of women are likely to have travelled from other catchment areas into ones with a health facility with full mother and child health services including delivery services, exceeding the target group sizes for those catchment areas.

The major limitation of immunisation data, which is considered further in the Discussion, is that they are based on administrative data, i.e. antigens recorded as delivered by health workers, and not usually (or in this case) based on coverage surveys of children [19]. Figures 1 and 2 illustrate a consistent upward trend in the number of clients or client episodes for a range of core HIV and non-HIV related mother and child health services; steady (PMTCT) or rapid (ART) increases in HIV service population coverage; and evidence that reproductive and child health population programme coverage levels were sustained or increased slightly. However, the question posed is: were these upward trends in HIV and non-HIV services happening in the *same *facilities?

**Figure 3:**
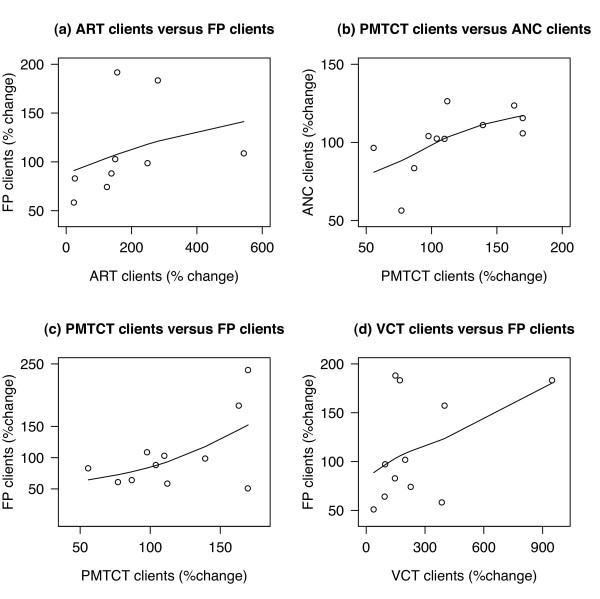
**Correlations of changes in services, 2005-2007**.

Table 3 reports Spearman rank intrafacility correlations in service trends between HIV and non-HIV services within the same facilities for a three year period: 2005 to 2007 inclusive. Percentage changes (increases or decreases) in numbers of clients/service episodes were calculated for each facility and Spearman rank correlations were measured, comparing pairs of services within each facility, to obtain summary measures of trend correlations (positive or negative) for each pair of services. Complete data were available on pairs of services, 2005-07, for between nine and twelve facilities. Comparisons between each of three HIV service trends (ART, PMTCT and VCT) and four non-HIV service trends (antenatal care, family planning, BCG and DPT3) produced 12 pairs of association (Table 3): nine showed positive correlations, two were negative and one was zero. Pairs of services where the Spearman rank correlation was greater than 0.3 are illustrated in scatter graphs in Figure 3. Plausible reasons for correlations are discussed later.

**Table 3:** Intrafacility correlations in service trends between HIV and non-HIV services, 2005-07 (Spearman rank correlations).

Facilities reporting clients for two services	Number of facilities	Spearman Rank Correlation (positive unless otherwise stated)
ART and Antenatal Clinic	9	0.22

ART and Family Planning Clinic	9	0.83

ART and DPT 3 vaccine	9	0.00

ART and BCG vaccine	9	0.27

PMTCT and Antenatal Clinic	12	0.50

PMTCT and Family Planning Clinic	12	0.33

PMTCT and DPT 3 vaccine	12	- 0.11

PMTCT and BCG vaccine	12	0.14

VCT and Antenatal Clinic	11	0.12

VCT and Family Planning Clinic	11	0.38

VCT and DPT 3 vaccine	11	0.15

VCT and BCG vaccine	11	- 0.30

The strongest rank correlation was between ART and family planning (Spearman rank coefficient = 0.83) in nine facilities that reported both sets of data between 2005 and 2007. This means that health facilities where the number of ART clients increased generally also experienced an increase in the numbers of family planning clients; and facilities that had greater increases in one service (ART) usually had greater increases in the other (family planning). It also illustrates that facilities that showed a fall in the numbers of ART clients, 2005-07, were more likely to see a fall in numbers of family planning clients. Family planning trends in seven of the non-ART providing facilities showed an increase between 2004 and 2007. Overall, family planning client numbers, which peaked in 2005, had fallen slightly by 2007 (Figure 2).

Positive associations between ART and antenatal clinic registrants (0.22) and between ART and BCG vaccinations (0.27) were weaker; and the correlation between ART and DPT3 was zero. Excluding one facility with a greater than 500% increase in the number of ART clients increased the latter correlation to 0.17; and a further exclusion of a facility with an upward DPT3 trend of >180% increased the correlation coefficient to 0.75. Such changes illustrate the effects of small sample sizes in the analysis and the sensitivity of the analysis to the effects of changes in just one or two facilities. Generally, removal of facilities with more extreme trends resulted in tighter correlations.

Positive correlations between trends in PMTCT and other reproductive health services were expected and were found (12 facilities in the analysis). There was a positive correlation between PMTCT and ANC attendance of 0.5 (Table 3). When one facility with an upward trend of 700% was excluded from the analysis, the correlation rose to 0.78. PMTCT and family planning client trends were also positively correlated (0.33) and there was a fairly strong positive correlation between VCT and family planning (0.38). Data were also collected on a range of other non-HIV services and support services, including a range of laboratory tests. Small numbers reduced the numbers of facilities available for trend analyses: for example, only district hospitals, larger clinics and large NGO facilities were providing laboratory services.

An assessment of stock maintenance for drugs and commodities yielded some relevant results (Table 4). Only 7% of facilities that stocked antiretroviral drugs (ARVs) reported stock-outs in the previous 12 months: 6% for first line ARVs (among 31 facilities stocking them) and 8% for second line (from 24 facilities). Stock-outs of important drugs or commodities for non-HIV priorities were significantly more frequent. These included Coartem - the new first line drug to treat Malaria (stock-outs had occurred in 50% of facilities) and Co-trimoxazole - a routine antibiotic (stock-outs in 28% of facilities) - both of which were significantly more common (p. <0.05) than were stock-outs of first and second line ARVs. Stock-outs of important commodities for the management of labour and delivery of infants - Oxytocin to accelerate labour (26%) and ergometrine for the management of postpartum haemorrhage (24%) - were also more common than for first line ARVs (p. < 0.05). Only two of four facilities that reported ARV stock-outs had experienced these for more than one month. Eleven of 19 (58%) stock-outs of Coartem were for greater than one month.

**Table 4:** Drugs and commodities normally stocked and stock-outs in 2007

Drug/commodity	Normally stocked	Stock-out in last 12 months	Stock out * greater than 1 month
ARV First-line**	31	2 (6.4%)	1 (50.0%)

ARV Second-line	24	2 (8.3%)	1 (50.0%)

Lamivudine	25	2 (8%)	0 (0.0%)

Rifampicin	33	7 (21.2%)	* 3 (42.9%)

Cotrimoxazole	39	11 (28.2%)	4 (36.4%)

Coartem	38	19 (50%)	* 11 (57.9%)

Male condom	37	6 (16.2%)	* 2 (33.3%)

Iron Tablets	39	8 (20.5%)	3 (37.5%)

Rehydration salts	38	7 (18.4%)	* 1 (14.3%)

Ergometrine	33	8 (24.2%)	4 (50.0%)

Oxytocin	23	6 (26.1%)	* 2 (33.3%)

IV giving test	37	6 (16.2%)	* 4 (66.7%)

Syringes	38	10 (26.3%)	* 3 (30%)

* Missing responses account for columns B and C not adding up to column A.

** Facility mapping identified 29 facilities providing ART. The pharmacy facility survey identifed a further 2 facilities stocking ARVs.

## Discussion

For too long, debates around the positive versus negative synergies and systemwide effects of HIV funding on other programme and disease control priorities, especially in sub-Saharan Africa with its multiple burdens of disease and fragile health systems, have ignored the need for evidence, or have relied on key informant interview studies [20]. These are valid methods, which we have used, but are not the method of choice for quantifying and comparing client numbers and trends for HIV and non-HIV priority services. They are vulnerable to bias, or at least wishful thinking, in the hotly contested arena where evidence of positive or negative effects of earmarked funding for specific diseases could influence donors' funding decisions. This paper does not provide *the *answer to the question: is successful scale-up of HIV services, which this paper confirms is happening in Zambia, having a beneficial or detrimental effect on other priority services. However, it does provide *an *answer, based on a small sample of facilities; and - more importantly - it provides an evaluation approach that needs to be replicated in larger samples and preferably on a national scale.

In facilities that had begun to rapidly scale-up HIV services and continued to provide other priority mother and child health services, the findings provide some support for the view that the former was having positive effects on the latter, at least with respect to antenatal care and family planning. How reliable is such evidence and how much weight can be placed on it? Firstly, there is a plausibility to it, or at least plausible hypotheses can be drawn from the findings, which need to be explored or tested. For example, there are obvious reciprocal connections, for example between PMTCT and antenatal care: one would expect (or at least want) that a facility that is offering PMTCT, as part of its antenatal care, would attract more pregnant women to attend for antenatal care. Also, antenatal care of women who come to a health facility when pregnant, is the main route to PMTCT. The linkage between PMTCT and antenatal care in Zambia has been demonstrated by Potter et al (2008) [15], who demonstrated improved quality of antenatal care in Lusaka facilities delivering PMTCT, as evidenced by trends in documented syphilis screening in pregnant women.

The strong positive associations of family planning client numbers with PMTCT, ART and VCT client numbers are interesting. Integrated services for family planning and HIV have been promoted over recent years [21-23]. A systematic review, which mainly focused on project-type interventions that sought to link family planning with VCT services, concluded that the evidence demonstrated the feasibility and effectiveness of integration [24]. The linkages are plausible and consistent with a move to provide integrated services to meet women's reproductive health needs, which dates back at least to the 1994 Cairo Conference on Population and Development.

We did not explore internal referral processes in these facilities, although others have reported on a programme that used antenatal care as an opportunity to initiate eligible women on ART [25]. One would expect (or at least want) that women who register for ART, perhaps postnatally on completion of PMTCT, would be referred to register for family planning services. This was the strongest correlation (Spearman coefficient = 0.83) in our analysis. One study estimated that the impact of PMTCT services would double in 14 high prevalence countries, by integrating family planning and PMTCT services [26]. A somewhat surprising finding in our analysis, which needs to be assessed in larger sample size quantitative analyses and explored in future explanatory mixed methods research (see Conclusion), was that the positive rank correlations of family planning client numbers with HIV service trends was partly due to downward trends in both sets of services in a minority of facilities and that numbers of family planning clients were increasing in facilities not delivering ART.

The significance of the study findings is that a population-based approach was used to select health facilities for the study. We did not select project facilities where a special primary health care approach was being used to ensure non-HIV priorities benefited in tandem with HIV service scale up. Nor was the study restricted to 'research facilities', although journal articles based on data from Lusaka indicate that a lot of ART and PMTCT research was going on in facilities that were included in our study [15,25]. Three of 11 PMTCT - antenatal care facility pairings in our study were from facilities outside of Lusaka, as were 4 of 12 PMTCT-family planning facility pairings. While enhanced integration may have been a programme effect, one might also expect the opposite where a programme supported by PEPFAR funding would prioritise HIV services at the expense of family planning services. A contextual factor that would help explain integrated services and positive correlations is Zambia's policy of "integration and scaling-up of the Prevention of Mother-to-Child Transmission of HIV and AIDS strategy into maternal and child health services" as a strategy to reach Millennium Development Goals (MDG) 4 and 5 [27].

There are some caveats or limitations to this study, before considering its broader significance. Firstly, it is based on a sample of three districts, though because of the inclusion of the capital city and another urban setting where early HIV scale-up was taking place, it did capture a high proportion of national ART scale up between 2004 and 2007. Also, only facilities that were identified in a May 2008 mapping exercise as providing ART were included, which would have excluded facilities that began to deliver ART subsequently [15]. Therefore representativeness cannot be assumed. However, it is the internal validity that is important, i.e. the findings do illustrate what was happening within the surveyed facilities.

The subsets of facilities where data were available to enable correlations to be measured resulted in small numbers (between 9 and 12 pairs for each analysis - see Table 3). The main reason for this was that the study aim was to demonstrate trends over time and the initiation of ART and PMTCT in 2006 in rural Mumbwa district meant that all of these facilities were excluded from the 2005-07 trend analysis. Consequently, Lusaka contributed most of the facilities to the analyses shown in Table 3 and Figure 3, ranging from eight of nine (for family planning and ART) to six of eleven facilities (for VCT and family planning). Hence, any hypotheses and assumptions around positive effects of HIV scale-up on other services have quite limited generalisability. Studies that utilise this approach need to be replicated with larger numbers and representative samples of facilities, capturing later years when HIV service roll-out has extended to more districts.

Secondly, routine health information collected by health workers has several limitations, though we would contend that analysis of such data is more important in the long run than conducting special surveys. Recording errors are possible at all stages from initial completion of reporting forms and client registers through to reporting at the national level, or as part of a research study [28,29]. Such problems have been identified in Zambia [30] and in our study four facilities reported different returns for numbers of clients registered for antenatal care and six for family planning, when comparing two sources of data - facility record reviews and district office data. Such errors reflect the lack of attention there has been to health facility data and reporting, while acknowledging that steps to improve routine data collection have been included in Zambia's 2009-2015 national information strategy [31].

Immunisation data returns are normally considered to be inherently inferior to population-based data, in that the former are based on numbers and types of vaccine antigens delivered, rather than on numbers and ages of children immunised. Coverage rates are then based on estimates of the target population, rather than on actual children identified in household surveys. The denominators (catchment populations) can be an underestimate of the size of the target group because children from other catchment areas that lack immunisation services may travel to facilities that offer such services. This can result in numerators (numbers of children immunised) that exceed the denominators (catchment target population sizes), as was found in this study. However, despite difficulties in interpretation that are commonly found when analysing routine vaccination data returns, these findings provide evidence that district immunisation programmes have continued to be delivered at a sustained level to the populations in the catchment areas of the three districts where HIV/AIDS scale up has been taking place. Intra-facility analysis did not demonstrate any consistent correlation between HIV and immunisation service trends.

The primary aim of the studies conducted under the umbrella of the Global HIV/AIDS Initiative Network (GHIN) was to assess the wider systems' effects of global HIV funding. The GHIN researchers recognised from the start the importance of measuring and acknowledging the primary aim and effects of the GHIs, which was to scale-up and reach more people with the HIV interventions that they needed. The findings on HIV scale up (Tables 1 and 2, and Figures 1 and 2) correspond with and confirm those reported by other researchers in Zambia. Our PMTCT data showed a similar trend to that of Stringer et al (2006) [16], which reported a decline in HIV positivity from 25.7% to 21.8% among pregnant women who were tested. This was not surprising, given that there would have been overlap in the data sources in Lusaka, which provided 81.4% of the pregnant women that were HIV tested in our study.

One of the GHIN objectives was also to assess access and equity effects, for example to assess if rural dwellers were also benefiting from interventions that were first rolled out in capital cities and other urban centres. In that respect, while Mumbwa rural district is not as difficult to reach as many parts of the Northern Province of Zambia, the findings show that once significant scale-up had started in Mumbwa (PMTCT in 2006 and ART in 2007) client numbers and coverage rose rapidly.

The wider significance of the findings in this study is that they illustrate the potential to derive useful evidence - for district as well as national programme managers - from routinely collected health facility data. Two approaches to collecting evidence on the performance of health systems have dominated in the last 10 years, one top-down and the other bottom- (or population-) up: the Health Metrics Network has carried out valuable work on the development of indicators and the Institute of Health Metrics has demonstrated the power of collating data and comparing performance across countries and regions [29,32]. Countries, such as Zambia, have followed this lead and have focused much of their efforts on aggregating data nationally and reporting to international fora such as the UNGASS [18].

The second approach has been bottom-up, where considerable efforts and funding has been allocated by donors to conducting household surveys, which - unlike health facility data - provide evidence of unmet need and health seeking behaviour, as well some evidence of services accessed [33,34]. However, as has been recognised by some commentators [30,35], insufficient attention has been paid to what is happening in the middle, that is at health facilities where disaggregated data need to be collected on performance, so as to identify good and poor performers and take action. In our initial 2007 survey, much effort was expended on collecting data on routine services (family planning, immunisations, antenatal care) directly from health facilities. Not uncommonly, data were missing because facilities had made data returns to the district office but had not retained copies at the health facility. This is symptomatic of what is fundamentally wrong about how health information systems (HIS) work in sub-Saharan Africa; or rather how they sometimes work for higher level planners (national, provincial and sometimes district), and do not work where they should work, which is at the health facility level.

In the 2008 survey we found that most of the routine non-HIV service data was available in disaggregated formats (disaggregated to individual health facilities) at district health offices. The non-HIV priority service data used in the paper were part of a functioning - if neglected - health information system. The senior researchers who were supervising the field work found little or no evidence that data were being analysed and acted upon at the facility, or even at the district level. One explanation for the non-use of data is the multiple burden of data collection at facility and district level in Zambia, due to parallel health information systems established to meet the information needs of global initiatives [36].

This paper demonstrates the feasibility of obtaining and analysing routinely collected data to illustrate the performance of non-HIV priority services in facilities where HIV services are scaling-up. Some findings should concern programme managers who have overall responsbility for the health services system. The reported availability in the previous 12 months of essential drugs for national priorities - malaria, bacterial infections and management of normal labor and obstetrical emergencies - was significantly poorer than for ARVs. While stock-outs were more frequent in the rural district, they were also (surprisingly) common in Lusaka. This suggests that, while AIDS funding *can *strengthen pharmaceutical management [13], non-HIV drug and commodity management had been less reliable in the previous year than it was for HIV. However, in the absence of trend data, one cannot infer any association between HIV and non-HIV pharmaceutical management.

The findings demonstrate upward trends in client numbers for non-HIV maternal and child health programmes, in three districts where HIV services were scaling-up. The analysis also demonstrates scale-up in reproductive health service client numbers (family planning and antenatal care), generally in the same facilities where HIV services were scaling up; and the rank correlation supports the interpretation that this was an aetiological link. However, the stated caveats mean that those who are seeking definitive evidence to conclude that investments in HIV benefit other service priorities should await more conclusive evidence. Interestingly, district childhood immunisations increased overall, but not in the facility catchment areas where HIV scale-up was happening.

## Conclusions

This paper has begun to answer some of the research questions raised by Rabkin et al (2009) [14], not only with respect to service linkages but also about the potential for using routine data for better evidence. What the paper does not do is to explain the trends. National planners can be encouraged about evidence that comprehensive reproductive health service delivery - which is part of Zambia's Vision 2030 strategy - boosts target population coverage. Researchers can justifiably interpret the findings in Lusaka as a reflection of the positive spin-offs from research [15], which may also be a Hawthorne Effect, whereby health workers who were being researched were motivated to increase their performance. However such interpretations are based on conjecture.

This study has helped to place some of the pieces of the jigsaw puzzle that comprises the health systems' effects of HIV funding. What is needed now is a replication of these intrafacility correlation analyses across a larger and more representative sample of facilities, including ones that are less subject to research, and across other countries. More important, though, is the need for explanatory studies that move beyond correlation studies to analyse the processes within facilities to understand and explain such trends. Sequential, explanatory mixed methods studies are needed, where facility staff are shown trend summaries and asked to provide explanations; and where patients/clients are interviewed, so as to understand how the availability and configuration of different services influence their health seeking behaviour. It is at the health facility level that most national disease control and population health targets will be achieved, or not.

## Competing interests

The authors declare that they have no competing interests

## Authors' contributions

RB led on study design, participated in data analysis and interpretation, prepared the first draft and participated in later drafts of the article. JS participated in data collection, data analysis and interpretation, and drafting of the article. AW participated in data collection, data analysis and interpretation, and drafting of the article. PD led on data analysis and participated in drafting of the article. PN participated in study design, data collection, data analysis and interpretation, and drafting of the article. All authors have seen and approved the final version.

## Pre-publication history

The pre-publication history for this paper can be accessed here:

http://www.biomedcentral.com/1471-2458/10/540/prepub
